# Reasons for discontinuing oral anticoagulation therapy for atrial fibrillation: a systematic review

**DOI:** 10.1093/ageing/afab024

**Published:** 2021-03-10

**Authors:** Jackie Buck, Julia Fromings Hill, Alison Martin, Cassandra Springate, Bikramaditya Ghosh, Rachel Ashton, Gerry Lee, Andrzei Orlowski

**Affiliations:** Faculty of Medicine and Health Sciences, University of East Anglia, Norwich Research Park, Norwich NR4 7TJ, UK; Cambridge University Hospitals NHS Foundation Trust, Cambridge CB2 0QQ, UK; Faculty of Medicine and Health Sciences, University of East Anglia, Norwich Research Park, Norwich NR4 7TJ, UK; Crystallise Ltd, Essex SS15 6SS, UK; Crystallise Ltd, Essex SS15 6SS, UK; Crystallise Ltd, Essex SS15 6SS, UK; Imperial College Health Partners, London NW1 2FB, UK; Adult Nursing, James Clerk Maxwell Building, King’s College London, London SE1 8WA, UK; The Health Economics Unit, West Bromwich, UK

**Keywords:** atrial fibrillation, oral anticoagulation, discontinuation, adherence, older people

## Abstract

**Introduction:**

Atrial fibrillation (AF) is the most common cardiac arrhythmia and can lead to significant comorbidities and mortality. Persistence with oral anticoagulation (OAC) is crucial to prevent stroke but rates of discontinuation are high. This systematic review explored underlying reasons for OAC discontinuation.

**Methods:**

A systematic review was undertaken to identify studies that reported factors influencing discontinuation of OAC in AF, in 11 databases, grey literature and backwards citations from eligible studies published between 2000 and 2019. Two reviewers independently screened titles, abstracts and papers against inclusion criteria and extracted data. Study quality was appraised using Gough’s weight of evidence framework. Data were synthesised narratively.

**Results:**

Of 6,619 sources identified, 10 full studies and 2 abstracts met the inclusion criteria. Overall, these provided moderate appropriateness to answer the review question. Four reported clinical registry data, six were retrospective reviews of patients’ medical records and two studies reported interviews and surveys. Nine studies evaluated outcomes relating to dabigatran and/or warfarin and three included rivaroxaban (*n* = 3), apixaban (*n* = 3) and edoxaban (*n* = 1). Bleeding complications and gastrointestinal events were the most common factors associated with discontinuation, followed by frailty and risk of falling. Patients’ perspectives were seldom specifically assessed. Influence of family carers in decisions regarding OAC discontinuation was not examined.

**Conclusion:**

The available evidence is derived from heterogeneous studies with few relevant data for the newer direct oral anticoagulants. Reasons underpinning decision-making to discontinue OAC from the perspective of patients, family carers and clinicians is poorly understood.

## Key points

- Persistence with OAC is crucial to prevent AF-related stroke and other comorbidities, but discontinuation rates are high
- Understanding the reasons why patients discontinue OAC is essential
- This systematic review showed diverse reasons including adverse events, clinical changes, physician advice, patient preference, hypersensitivity, and medication interactions
- However, studies are highly heterogeneous and further research is needed compare patients’ and physicians’ motivations for discontinuing OAC

## Introduction

Atrial fibrillation (AF) is a cardiac rhythm disorder that increases mortality and morbidity due to thromboembolic events such as stroke and myocardial infarction [1,2]. The risk of mortality and morbidity due to AF increases with age, with incidence being highest among people older than 75 years and mortality being around 15% in these patients [1,3]. The prevalence of AF has risen particularly in higher-income countries [4]. The projected prevalence of AF is expected to triple in the next 10–20 years, possibly reaching around 9 million in the USA by 2030 [5] and 18 million in Europe by 2060 [6].

International guidelines, such as those from the American Heart Association/American College of Cardiology/Heart Rhythm Society [7] and the European Society of Cardiology [8], and the UK national guidelines from the National Institute of Health and Care Excellence [9] recommend the use of oral anticoagulation (OAC) as a preventive measure against stroke. Use of OAC significantly reduces the chances of thromboembolic cardiovascular events, such as stroke, and mortality in patients with non-valvular AF [10].

Two main groups of oral anticoagulant drugs are used to treat AF: vitamin K antagonists ([VKAs] most commonly warfarin) and non-VKAs, also known as direct oral anticoagulants (DOACs), including dabigatran, apixaban, rivaroxaban and edoxaban. A meta-analysis of the efficacy of OAC for stroke prevention in AF concluded that DOACs were more effective and cost-effective than warfarin [11]. However, despite being highly effective in reducing and preventing stroke and embolism in clinical trials, in real-world settings, the efficacy of treatments depends greatly on medication being prescribed and taken as recommended. Persistence rates with OAC vary but are typically around 15–20% [12,13]. The patterns of persistence and adherence rates also differ by drug type, with studies indicating differences in patients’ preferences for warfarin or DOACs [14,15].

Discontinuing treatment can have negative consequences, including increased disease burden. In accordance, the risk of hospitalisation and higher total health-care costs rise [2,16]. Understanding the reasons why patients discontinue OAC is essential to tackling these issues.

The aim of this study was to determine the reasons that OAC is permanently discontinued, with a focus on patient, clinician and family/carer perspectives.

## Methods

### Search strategy

We conducted a systematic literature search to identify articles published between 2000 and 2019 that reported on factors influencing discontinuation of OAC in AF. The search strategy was developed in collaboration with an academic librarian. The search terms were generated from combinations of ‘atrial fibrillation’, ‘oral anticoagulants’ and ‘discontinuation’. The full search strategies for Medline (via EBSCO), EMBASE, Cochrane Database of Systematic Reviews, PsycINFO and OpenGrey are shown in the Supplementary data available in *Age and Ageing* online (pp 1–5). These were adapted for searches in CINAHL, ASSIA, Scopus, COPAC, Ethos and Proquest. Potentially relevant articles were identified by title and abstract only. Manual searches of the reference lists of relevant review papers and lateral searches of reference lists from all included papers were also carried out.

### Data synthesis

Titles, abstracts and full texts (if retrieved) were independently screened by two members of the review team. Papers were included if they were written in English and described reasons for discontinuation of OAC in people with AF. Studies that described the temporary cessation of OAC or reasons for switching oral anticoagulant drugs were excluded unless they also described reasons for permanent discontinuation.

Full inclusion and exclusion criteria are shown in the Supplementary data available in *Age and Ageing* online (p 6). Briefly, data were classified by reason for discontinuation: decision by the patient, decision by the physician, due to adverse events, due to frailty, no AF and other reasons. Additionally, papers were classified according to the inter-relationships between reasons.

Discrepancies in screening decisions were discussed within the team. Any study for which the abstract did not make relevance clear was retrieved and the full text was screened.

### Data quality

Studies were quality assessed for risk of bias with Gough’s weight of evidence framework [17]. Quality of evidence was assessed according to three main dimensions:

- the quality of the execution of the study (irrespective of the review question);
- the appropriateness of the research design for answering the review question; and
- the appropriateness of the study focus in the context of the review question.

To assess the quality of the execution of the study (dimension A) we used an adapted version of the NHLBI quality assessment tool for observational cohort and cross-sectional studies (https://www.nhlbi.nih.gov/health-topics/study-quality-assessment-tools). Two researchers weighted each study independently and differences were reconciled by discussion.

## Results

**Figure 1:**
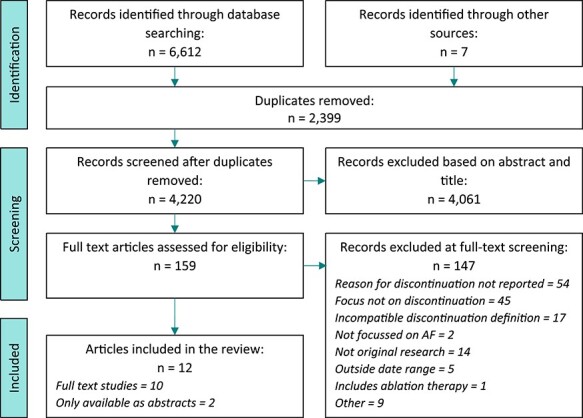
PRISMA flowchart.

The PRISMA flow diagram (Figure 1) summarises the article selection process. After removal of duplicate publications and application of exclusion criteria, 12 of 6,619 identified sources met all the inclusion criteria—10 full-text articles and 2 conference abstracts [18–29].

### Quality of the evidence

The results of the quality assessment for Gough’s dimension A are shown in Table 1. The quality of studies was generally consistent. The areas of poorest quality were sample size justification, levels of the exposures assessed and the handling of confounding variables.

**Table 1:** Quality of the execution of the study

Study	Was the research question clearly stated?	Was the study population clearly specified and defined?	Was recruitment and screening the same or similar across groups?	Was a sample size justification provided?	Was the timeframe sufficient?	Were different levels of exposure examined?	Were the exposure measures defined, valid, reliable and consistent?	Were the outcome measures defined, valid, reliable and consistent?	Were confounding variables considered and outcomes adjusted accordingly?
Borg Xuereb 2016 [18]	Yes	Yes	Yes	Yes	Yes	Yes	Yes	Yes	No
Gumbinger 2015 [19]	Yes	Yes	Yes	No	Yes	Yes	Yes	Yes	No
Jackson II 2018 [20]	Yes	Yes	Yes	No	Yes	No	Yes	Yes	No
O’Brien 2014 [21]	Yes	Yes	Yes	No	Yes	No	Yes	Yes	No
Paquette 2017 [22]	Yes	Yes	Yes	No	Yes	No	Yes	Yes	No
Paquette 2018 [23]	Yes	No	Yes	Unclear	Yes	Unclear	Yes	Yes	No
Park 2019 [24]	Yes	Yes	Yes	No	Yes	No	Yes	Yes	No
Renner 2019 [25]	Yes	No	Yes	Unclear	Yes	Unclear	Yes	Yes	No
Naganuma 2017 [26]	Yes	Yes	Yes	No	Yes	Yes	Yes	Yes	No
Shiga 2015 [27]	Yes	Yes	Yes	No	Yes	Yes	Yes	Yes	No
Bertozzo 2016 [28]	Yes	Yes	Yes	No	Yes	No	Yes	Yes	No
Ho 2014 [29]	Yes	Yes	Yes	No	Yes	Yes	Yes	Yes	No

The foci and objectives in most studies related to discontinuation rates, risk factors for discontinuation of treatment and/or the reasons for discontinuations. Overall, the studies provided moderate appropriateness to answer the review question (Supplementary data available in *Age and Ageing* online, p 7). Two studies had qualitative study designs and asked specifically for reasons underlying discontinuation among other data gathered [18,19]. No studies focussed solely on the reasons reported by patients for permanently discontinuing OAC.

### Summary of included studies

**Figure 2:**
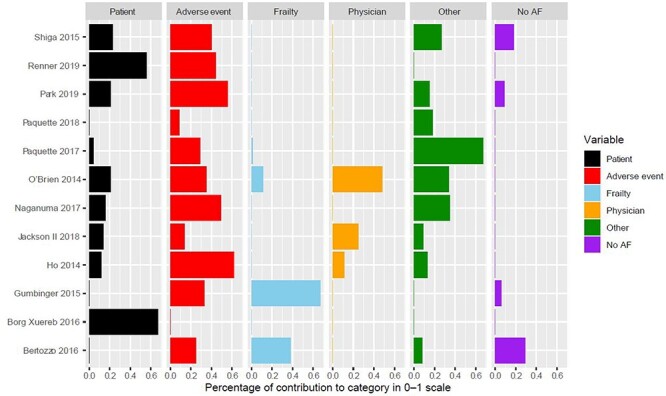
Classification of data.

**Figure 3:**
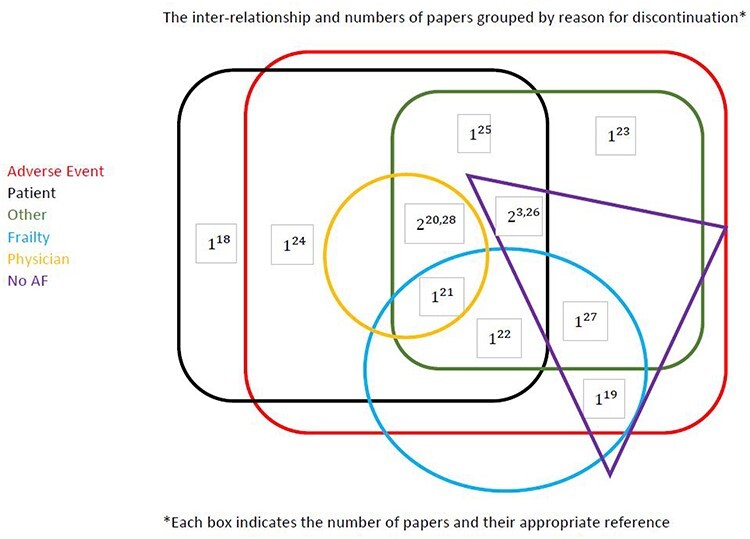
Inter-relationship of reasons for discontinuation.

Of the 12 studies included in the review (Table 2), most provided reasons related to the categories discontinuation due to patient’s choice, adverse events and other reasons (Figure 2). Two reported discontinuation data from patient surveys and interviews [18,19], four reported data from clinical registries of patients with AF [21–23] and six reported data obtained from retrospective reviews of patients’ charts and hospital records [24–29]. Most studies provided information supporting inter-relationships between reasons (Figure 3).

**Table 2:** Summary of included studies

Study	Design	Setting	Population	Oral anticoagulant drugs evaluated	Follow-up	Definition of discontinuation	Discontinuation rates	Source of discontinuation data
Studies reporting reasons for discontinuations provided directly by patients								
Borg Xuereb 2016 [18]	Qualitative	Hospital admissions in the UK	11 adult patients with AF	Warfarin	Not applicable	Not applicable	Not applicable	Semi-structured interviews
Gumbinger 2015 [19]	Qualitative	Hospital, Germany	139 patients with TIA, IS and AF	Various drugs	15 (±1) months	Permanent discontinuations: definition not reported; others defined as treatment interruptions	Permanent discontinuations: 15.1% (*n* = 109)	Semi-structured interviews
Studies reporting reasons for discontinuations selected by physicians from pre-specified lists								
Jackson II 2018 [20]	Retrospective cohort	Clinical registry including data from 176 sites in the USA	7,150 patients with AF	Dabigatran, Warfarin	6 months and 1 year	Discontinuation of the drug at 6 or 12 months for any reason	6 months: Dabigatran = 23.8% Warfarin = 10.5% 12 months: Dabigatran = 36.8% Warfarin = 17.3% Between groups *P* < 0.0001	ORBIT-AF prospective registry data (possible data duplication with O’Brien 2014)
O’Brien 2014 [21]	Retrospective cohort	Clinical registry including data from 176 sites in the USA	7,121 patients with AF	Warfarin	1 year	Patients not receiving OAC at 6- or 12-month follow-up	Overall = 11% 10.1% of those on warfarin at baseline 17.1% starting warfarin during the study period	ORBIT-AF prospective registry data (possible data duplication with Jackson II 2018)
Paquette 2017 [22]	Prospective cohort	Clinical registry in 44 countries across Asia, Europe, North America, Latin America, and Africa/Middle East	2,932 patients with AF	Dabigatran	2 years	Interruption of therapy for >30 days	Without switching: 14.9%	GLORIA-AF prospective registry data (possible data duplication with Paquette 2018)
Paquette 2018 [23]	Prospective cohort	Clinical registry in 44 countries across Asia, Europe, North America, Latin America, and Africa/Middle East	4,873 patients with AF	Dabigatran	2 years	Interruption of therapy for >30 days	Without switching: 14.1%	GLORIA-AF prospective registry data (possible data duplication with Paquette 2017)
Studies reporting reasons for discontinuations recorded on medical records								
Park 2019 [24]	Prospective cohort	Tertiary hospitals in Korea	866 patients with NVAF and no history of bleeding	Various VKA	1 year	Permanent discontinuation without resumption	Without switching: 6.81%	Medical records
Renner 2019 [25]	Retrospective cohort	Hospital, USA	319 patients with AF	Dabigatran, Rivaroxaban, Apixaban, Edoxaban	Not reported	Not reported	Overall 14%	Medical records
Naganuma 2017 [26]	Retrospective cohort	Hospital, Japan	819 patients with NVAF	Dabigatran, Rivaroxaban, Apixaban, Warfarin	2 years	Permanent discontinuation of drug prescription and the physician’s mention of drug discontinuation in the medical record	Dabigatran = 6.0% Rivaroxaban = 4.5% Apixaban = 4.5% Warfarin = 5.7%	Medical records (possible data duplication with Shiga 2015)
Shiga 2015 [27]	Retrospective cohort	Hospital, Japan	601 patients with NVAF	Dabigatran, Rivaroxaban, Apixaban, Warfarin	2 years	Discontinuation of drug prescription, and physician’s mention of drug cessation on the medical record, including switching treatments	Permanent discontinuation without switching: Dabigatran = 5.7% Rivaroxaban = 5.6% Apixaban = 5.9% Warfarin = 12%	Medical records (possible data duplication with Naganuma 2017)
Bertozzo 2016 [28]	Retrospective cohort	Hospital, Italy	798 older patients (≥80 years of age) with NVAF	Warfarin	≥6 months (mean 29 [±18.7] months)	≥180 consecutive days without warfarin treatment in which there were no sequential INR measurements	Discontinuation: 18.5% of 798 included in the analysis Of 888 screened 22% discontinued	Medical records
Ho 2014 [29]	Retrospective cohort	Hospital, China	467 patients with AF	Dabigatran	Mean 16 (±10) months	Permanent discontinuation without resumption (unclear if this includes switching treatment)	21.6%	Medical records

NVAF = non-vascular atrial fibrillation.

Two studies reported data from registries: outcomes registry for better-informed treatment of atrial fibrillation (ORBIT-AF) [20,21] and Global Registry on Long-Term Oral Anti-thrombotic Treatment In Patients With Atrial Fibrillation (GLORIA-AF) [22,23]. ORBIT-AF is a national US registry and GLORIA-AF is an international registry containing information from 44 countries. Both record patients with newly diagnosed AF initiated on OAC. Most of the data are from Europe and North America due to the relevant drugs being more widely available in these regions. Both registries included a pre-specified list of reasons for discontinuations of treatment from which physicians may select one or multiple options. Two studies included interviews with patients, conducted in the UK [18] and Germany [19], and six employed retrospective chart reviews, one in Korea [24], one in the USA [24], two in Japan [26,27], one in Italy [28] and one in China [29]. The two Japanese studies were conducted separately but in the same department at Tokyo Womenʼs Medical University Hospital. One research group was responsible for the two ORBIT-AF registry studies, as was one group for the two Tokyo studies. It is likely, therefore, that some data were duplicated.

All studies focussed on adults with AF. One study specifically assessed very old patients (≥80 years) [28] and another included patients who had AF and experienced transient ischaemic attack or ischaemic stroke (IS) [19]. Sample size varied substantially across studies, from 11 to 139 participants in the qualitative studies [18,19], from 300 to 900 in the retrospective chart review studies [24–29] and from 2,900 to 7,200 in the registry studies [20–23].

Most studies evaluated outcomes relating to dabigatran and/or warfarin. Two retrospective chart review studies reported data on patients treated with dabigatran, rivaroxaban, apixaban or warfarin [26,27], and one on patients treated with dabigatran, rivaroxaban, apixaban or edoxaban [25]. Three studies reported results for patients treated only with dabigatran [22,23,29] or only warfarin [18,21,30], one study reported on discontinuations from both warfarin and dabigatran [20], and two studies did not specify which oral anticoagulant drugs patients had received [19,24].

### OAC discontinuation rates

Heterogeneity in the sample sizes and follow-up durations made it difficult to compare OAC discontinuation rates directly. In addition, two studies provided no definition of discontinuation [18,25] and did not make it clear whether discontinuation included patients who switched to an alternative OAC [20,28].

Follow-up periods varied but were generally 1–2 years (Table 2). Discontinuation rates reported for warfarin treatment or unspecified VKAs at 1 year ranged from 6.8 to 17.3% and at 2 years from 5.7 to 12% [26–28] (Table 2). At the longest reported follow-up (mean 29 months), the rate was 18.5% [28]. In contrast, discontinuation rates for dabigatran were much higher, being 36.8% at 1 year [20], although rates fell over time, to 21.6% after a mean follow-up of 16 months [29] and ranging from 6.0 to 14.9% by 2 years [22,23,26,27], which were similar to those for warfarin treatment (Table 2).

Three studies reported discontinuation rates for other DOACs. In an abstract by Renner and colleagues [25], the overall discontinuation rate was 14% for dabigatran, rivaroxaban, apixaban and edoxaban combined, but the follow-up period was not reported. In the two registry studies conducted in Japan [26,27], contrasting results were seen. In the study by Shiga *et al*. [27], at 2 years of follow-up, discontinuation rates were highest with warfarin and were roughly halved for apixaban, rivaroxaban and dabigatran [27] (Table 2). In contrast, discontinuation rates at 2 years reported in Naganuma *et al.* [26] were highest for dabigatran followed by warfarin, apixaban and rivaroxaban, although values range only from 4.5 to 6.0% (Table 2). The difference in discontinuation rates for warfarin reported by these two studies was unexpected given that the data were obtained from the same database, from approximately the same time period, with the same inclusion criteria and definitions of discontinuation. This inconsistency could highlight issues with heterogeneity in the data.

### Reasons for discontinuation

The reasons for discontinuation of OAC were generally not reported in detail and the relative importance of different reasons varied across the studies. All reasons and rates for discontinuations are provided in the Supplementary data available in *Age and Ageing* online (pp 8–12).

Two small studies reported data obtained directly from interviews with and communications from patients and physicians [18,19]. The larger study included 139 patients in whom OAC was indicated after myocardial infarction or transient ischaemic attack (101 new prescriptions and 38 continuations); 85 should have been taking OAC at 15 months of follow-up. Among these, discontinuations were recorded in 3.6% due to a decline in functional status, 2.8% due to increased risk of falling and the same for bleeding events, 2.2% due to a diagnosis of dementia, and 0.8% due to no evidence of AF on a follow-up ECG [19]. No additional qualitative analysis of motivations for discontinuation was presented.

In the smaller study, which explored experiences of patients and physicians during consultations for AF in 11 patients, three patients discontinued warfarin [18]. Two cited anticoagulation monitoring as the reason. One reported that his employer was unsympathetic to the need for regular clinic visits. The second patient felt that regular monitoring was incompatible with his desire to travel while he felt he was healthy enough to do so.

Reasons for discontinuation from the database and registry studies were mostly reported only in general terms with very few details and minimal interpretation of the motivations. Specific reasons for discontinuation were most commonly reported to be adverse events, in particular bleeding and gastrointestinal events, while most other reasons were summarised as patients’ preferences or physicians’ choices.

Bleeding complications were common reasons for OAC discontinuation, with rates of 3–25% for warfarin [20,21,26–28], 5.5% for unspecified VKAs [24] and 20–22% for any OAC [19,25]. This reason was also given frequently for discontinuation of DOACs (3.4–9.7% for dabigatran [20,21,26,27], 5.7–10.1% for apixaban [26,27] and 16.8–24.5% for rivaroxaban [26,27]).

Discontinuations due to gastrointestinal adverse events, symptoms and ‘upset’ were most common for dabigatran (6.5–13.8%) and rivaroxaban (6.8–13.5%) [20,21,26,27,29]. By comparison, discontinuation rates for this reason were 0–6.7% for apixaban [26,27] and 0.4–4.8% for warfarin [20,21,26–29]. Discontinuation rates for all or any adverse events varied from 6.2 to 62.4% for dabigatran [22,23,26,27], from 49.9 to 56.6% for apixaban [26,27], from 36.7 to 46.0% for rivaroxaban [26,27] and from 18.2 to 31.8% for warfarin [26,27]. Wide variations were also found in the reported rates of discontinuations due to ‘other’ adverse events; the events in this category were not specified.

Frailty, specifically regarding an increased risk of falling and low life-expectancy, was listed as a reason in three studies and was associated with 11–38% of discontinuations [19,21,28]. Cost was listed as a reason in three separate studies: 38% due to drug costs [25] and 1–2% due to treatment costs and financial concerns [22,29].

While patients’ desires, preferences and refusals to continue with treatment were commonly reported, the underlying motivations were not widely analysed. Among 366 patients receiving dabigatran in one study, patient concerns were listed in 3.3% of discontinuations, with specific reasons of dosing frequency being given in 2%, side effects in 1%, financial issues in 0.3% and the need for monitoring in 0.3% [29]. Other studies reported that undefined patients’ preferences were associated with 5.7–46.3% of discontinuations [20,21,25–27].

Non-adherence is a separate issue from discontinuation, as many patients fail to take all their prescribed doses of a medication but do not stop entirely. However, one study found that 3.4% of discontinuations of warfarin were due to physicians’ concerns about poor adherence [28]. Physicians in another study reported that 4.7% of warfarin discontinuations were due to poor adherence to drugs and/or monitoring [21].

Other reasons reported by only a few of the studies included bruising, dementia, hypersensitivity, comorbidities, interactions with concomitant medication, abnormal laboratory data and worsened renal function. Only one study included social issues (e.g. alcohol misuse) and occupational risk as reasons and associated them with only 1.6% of discontinuations [22].

## Discussion

OAC discontinuation has a considerable impact on the negative consequences of AF, including increased morbidity, mortality and related health-care costs [16]. It is therefore important to understand the reasons why oral anticoagulant drugs are discontinued in patients with AF. The studies included in this review reported various and wide-ranging reasons underlying discontinuations, including adverse events, clinical and biochemical changes, advice from the physician, patients’ preferences and concerns, comorbidities, cost, hypersensitivity and interactions with concomitant medication.

The most commonly reported reason for OAC discontinuation was adverse events. Within this category, experiencing or being deemed to be at high risk of bleeding events and gastrointestinal adverse events were the most frequent reasons. Although DOACs are associated with reduced risk of bleeding, the discontinuation rate for rivaroxaban was high (16.8–24.5%. Dabigatran and rivaroxaban were most often discontinued for gastrointestinal effects. However, the proportion of patients stopping OAC due to bleeding risk ranged from 3 to 25% across all drugs assessed. This heterogeneity in results makes it is difficult to draw many firm conclusions on, for example, the thresholds for discontinuation.

One study included in our review reported a qualitative analysis of patients’ motivations for discontinuing treatment and highlighted the issue of disease management [18]. Regular monitoring of warfarin treatment interfered with work and lifestyle choices. Unfortunately, though, only two patients were quoted and no other studies specifically explored monitoring requirements as a factor in discontinuation. This factor warrants further investigation.

There were several limitations of the included studies that could have affected this review’s conclusions. First, substantial missing data were noted. The reason for discontinuing treatment was listed as unknown in 3–10% of cases in studies of hospital chart reviews [24,28,29]. A possible explanation for this uncertainty is incomplete record keeping. This incomplete representation of reasons caused a significant problem when interpreting the study results and potentially masked confounding factors that could affect the findings. For example, if physicians were more likely to record the reasons when they were related to medical complications or concerns (e.g. adverse events or abnormal laboratory data) than patients’ preferences, the data would be skewed. Second, the definition of discontinuation varied and/or was unclear. Such information could be useful to investigate whether certain factors affect the likelihood of restarting OAC, such as temporary stoppage of OAC due to other treatments or the time elapsed since discontinuation. Third, even though we specifically looked for studies that and included aspects such as the roles of family and networks, level of education, and effects on employment, only one [21] explored broader factors for discontinuation and these were briefly mentioned by only two patients. It would be useful to expand the designs of future studies to include such factors, as this could provide much-needed understanding of why patients discontinue OAC. Finally, substantially more data are available for discontinuation of warfarin versus all DOACs, but especially rivaroxaban, apixaban, and edoxaban. The small number of results for some DOACs suggested heterogeneity within this class, but forming reliable conclusions is not yet possible. The similarities and differences for the DOACs need to be more clearly understood. Because of the lack of conformity of the data, it was not possible to undertake any meta-analyses.

In contrast to discontinuation, many studies report on treatment adherence. Although not the same as discontinuation, non-adherence could be a possible cause of permanent discontinuation of OAC. Achievable strategies, such as improving patients’ knowledge, shared decision-making and ensuring patients feel their preferences are taken into account by physicians, can increase OAC adherence [30–32]. Whether these approaches can also affect treatment discontinuations needs to be assessed.

## Conclusions

The occurrence and increased risk of adverse events, particularly bleeding and gastrointestinal events, were the most common reasons for discontinuing OAC. Of note, though, there are no obvious or reliable differences between the individual OAC drugs or drug classes (VKAs vs. DOACs). Importantly, the available evidence is derived from heterogeneous studies with few relevant data being available for the newer DOACs. Future studies of patients’ and physicians’ motivations for discontinuing OAC will be crucial to understand what seems to be a wide variety of underlying reasons and how to address them. Developing the evidence base for targeted interventions to reduce discontinuation of these therapies is vital so that optimal anticoagulation to prevent stroke can be achieved in this cohort.

## Supplementary Material

aa-20-1464-File002_afab024
