# Supplementary material for: Reasons for discontinuing oral anticoagulation therapy for atrial fibrillation: a systematic review
Source: Age Ageing. 2021 Mar 10;50(4):1108–17. doi: 10.1093/ageing/afab024 (PMC8839858; doi:10.1093/ageing/afab024)
Supplement: aa-20-1464-File002_afab024 [file aa-20-1464-file002_afab024.docx]

**Reasons for discontinuing oral anticoagulation therapy for atrial fibrillation: a systematic review**

**SUPPLEMENTARY DATA**

Table 1. MEDLINE search

| **Search ID** | **Search String (Limits: English, publication date 2000-2019)** | **Hits** |
| --- | --- | --- |
| 1 | atrial fibrillation'/mj | 27680 |
| 2 | atrial fibrillation'/exp OR 'atrial fibrillation' | 71,408 |
| 3 | atrial fibrillation':ab | 37094 |
| 4 | 1 OR 2 OR 3 | 71,408 |
| 5 | anticoagulant agent'/exp OR 'anticoagulant agent' | 289361 |
| 6 | anticoagulant agent'/mj | 13,517 |
| 7 | anticoagulation'/exp OR 'anticoagulation' | 40225 |
| 8 | anticoagulation'/mj | 4,333 |
| 9 | anticoagula*:ab | 47635 |
| 10 | (anticoagula* NEAR/1 therap*) | 49099 |
| 11 | noac:ab | 791 |
| 12 | doac:ab | 695 |
| 13 | antithrombin:ab | 6537 |
| 14 | warfarin:ab | 13691 |
| 15 | vit* k antagonist*':ab | 4093 |
| 16 | vka:ab | 1365 |
| 17 | 5 OR 6 OR 7 OR 8 OR 9 OR 10 OR 11 OR 12 OR 13 OR 14 OR 15 OR 16 | 307713 |
| 18 | patient compliance'/exp OR 'patient compliance' | 90945 |
| 19 | adheren* | 112671 |
| 20 | complian* | 161060 |
| 21 | concordance'/exp OR 'concordance' | 31628 |
| 22 | concordan* | 45360 |
| 23 | persist* | 45737 |
| 24 | discontinu* | 69912 |
| 25 | implemen* | 325729 |
| 26 | drug taking' | 1006 |
| 27 | medicine optimi*' | 921033 |
| 28 | 18 OR 19 OR 20 OR 21 OR 22 OR 23 OR 24 OR 25 OR 26 OR 27 | 261209 |
| 29 | 4 AND 17 AND 28 | 3726 |

Table 2. EMBASE search

| **Search ID** | **Search String (Limits: English, publication date 2000-2019)** | **Hits** |
| --- | --- | --- |
| 1 | atrial fibrillation'/mj | 49553 |
| 2 | 'atrial fibrillation'/exp OR 'atrial fibrillation' | 128493 |
| 3 | atrial fibrillation':ab | 83158 |
| 4 | 1 OR 2 OR 3 | 128493 |
| 5 | anticoagulant agent'/exp OR 'anticoagulant agent' | 414255 |
| 6 | anticoagulant agent'/mj | 15343 |
| 7 | anticoagulation'/exp OR 'anticoagulation' | 66155 |
| 8 | anticoagulation'/mj | 8235 |
| 9 | anticoagula*:ab | 86318 |
| 10 | (anticoagula* NEAR/1 therap*) | 59916 |
| 11 | noac:ab | 2347 |
| 12 | doac:ab | 1850 |
| 13 | antithrombin:ab | 9557 |
| 14 | warfarin:ab | 26127 |
| 15 | vit* k antagonist*':ab | 7389 |
| 16 | vka:ab | 3322 |
| 17 | 5 OR 6 OR 7 OR 8 OR 9 OR 10 OR 11 OR 12 OR 13 OR 14 OR 15 OR 16 | 444044 |
| 18 | patient compliance'/exp OR 'patient compliance' | 111017 |
| 19 | adheren* | 163308 |
| 20 | complian* | 224265 |
| 21 | concordance'/exp OR 'concordance' | 49780 |
| 22 | concordan* | 70792 |
| 23 | persist* | 393151 |
| 24 | discontinu* | 131828 |
| 25 | implemen* | 3760111 |
| 26 | drug taking' | 1354 |
| 27 | medicine optimi*' | 45 |
| 28 | 18 OR 19 OR 20 OR 21 OR 22 OR 23 OR 24 OR 25 OR 26 OR 27 | 1232700 |
| 29 | 4 AND 17 AND 28 | 7600 |

Table 3. Cochrane Database of Systematic Reviews search

| **Search ID** | **Search String** | **Hits** |
| --- | --- | --- |
| #1 | MeSH descriptor: [Atrial Fibrillation] explode all trees | 4025 |
| #2 | 'atrial fibrillation':ab | 8922 |
| #3 | atrial fibrillation | 11782 |
| #4 | #1 OR #2 OR #3 | 11782 |
| #5 | MeSH descriptor: [Anticoagulants] explode all trees | 4381 |
| #6 | 'anticoagulant':ab | 3731 |
| #7 | anticoagulan* | 10435 |
| #8 | anticoagulant agent | 1715 |
| #9 | anti coagulation | 199 |
| #10 | NOAC OR "NOAC":ab | 250 |
| #11 | novel anticoagulant | 24 |
| #12 | DOAC OR "DOAC":ab | 164 |
| #13 | direct oral anticoagulants | 244 |
| #14 | MeSH descriptor: [Warfarin] explode all trees | 1544 |
| #15 | warfarin:ab OR warfarin | 4886 |
| #16 | vitamin k antagonist | 576 |
| #17 | MeSH descriptor: [Vitamin K] explode all trees | 545 |
| #18 | 'vit* k antagonist*':ab | 1239 |
| #19 | antithrombin:ab | 1537 |
| #20 | #5 OR #6 OR #7 OR #8 OR #9 OR #10 OR #11 OR #12 OR #13 OR #14 OR #15 OR #16 OR #17 OR #18 OR #19 | 15165 |
| #21 | MeSH descriptor: [Patient Compliance] explode all trees | 11316 |
| #22 | patient compliance | 19606 |
| #23 | complian* | 46130 |
| #24 | adheren* | 29322 |
| #25 | concordance OR concordan* | 4319 |
| #26 | persist* | 38607 |
| #27 | discontinu* | 35056 |
| #28 | implemen* | 36566 |
| #29 | drug taking* | 136 |
| #30 | 'medicine optimi*' | 4348 |
| #31 | #21 OR #22 OR #23 OR #24 OR #25 OR #26 OR #27 OR #28 OR #29 OR #30 | 164816 |
| #32 | #4 AND #20 AND #31 with Publication Year from 2000 to 2019, in Trials | 526 |

Table 4. OpenGrey search

| **Search ID** | **Search String** | **Hits** |
| --- | --- | --- |
| 1 | (atrial fibrillation) AND ("anticoagulants" OR "Oral anticoagulants" OR "NOAC" OR "DOAC" OR "antithrombin" OR "warfarin" OR "Vitamin K antagonist") | 21 |
| 2 | applying limits: language english & year 2000-2019 | 5 |

Table 5. PsycINFO search

| **Search ID** | **Search String (Limits: 2000-2019)** | **Hits** |
| --- | --- | --- |
| 1 | atrial fibrillation | 1233 |
| 2 | anticoagulant* OR "NOAC" OR "DOAC" OR "antithrombin" OR "Warfarin" OR "Vitamin K antagonist" OR vka | 1382 |
| 3 | "patient compliance" OR "complian*" OR "adheren*" OR "persist*" OR "discontinu*" OR "concordan*" OR "medicine optimi*" | 124599 |
| 4 | 1 AND 2 AND 3 | 61 |

Table 6. Inclusion/exclusion criteria for study selection

| **Criterion** | **Inclusion criterion** | **Exclusion criterion** |
| --- | --- | --- |
| Disease | Atrial fibrillation (AF) | Venous thromboembolism (deep vein thrombosis, pulmonary embolism, stroke, transient ischaemic attacks etc)  Mixed cardiovascular disease populations where <80% have AF |
| Population | Adults  Caregivers of patients taking OACs for AF  Healthcare professionals involved in the care of patients with AF taking OACs | Healthy volunteers  Animals, laboratory samples  Children or adolescents  Mixed age populations where <80% are adults |
| Interventions | Any oral anticoagulant eg  Warfarin  Apixaban  Dabigatran  Edoxaban  Rivaroxaban  Vitamin K antagonist | Antiplatelet drugs eg aspirin, clopidogrel  Heparins  Injected anticoagulants |
| Outcomes | Reasons for stopping OACs  Patient/caregiver/ healthcare professional views about OACs  Barriers and facilitators to continuing OACs | Discontinuation rates where no reasons are given |
| Study methodology | RCTs  Other comparative clinical trials  Single arm clinical trials  Database or registry studies  Qualitative studies  Other observational studies  Systematic reviews of relevant studies | Narrative reviews  Opinion pieces/ editorials/ letters  Case studies  Study protocols with no results reported  Conference abstracts with no useful data  Conference abstracts where a full text publication has been identified |
| Language | English language only |  |
| Publication date | Entire review: 2010 to 2019  This update: 2018 to 2019 |  |

Table 7. Appropriateness of the included studies

| **Study** | **Appropriateness of the research design for answering the review question** | **Appropriateness of the study focus for the review** |
| --- | --- | --- |
| Borg Xuereb 2016 (18) | High | Moderate |
| Gumbinger 2015 (19) | High | Moderate |
| Jackson II 2018 (20) | Moderate | Moderate |
| O'Brien 2014 (21) | Moderate | Moderate |
| Paquette 2017 (22) | Moderate | Moderate |
| Paquette 2018 (23) | Moderate | Moderate |
| Park 2019 (24) | Moderate | Low |
| Renner 2019 (25) | Moderate | Moderate |
| Naganuma 2017 (26) | Moderate | Low |
| Shiga 2015 (27) | Moderate | Moderate |
| Bertozzo 2016 (28) | Low | Moderate |
| Ho 2014 (29) | Moderate | Moderate |

*Table 8. Reasons for discontinuation of treatment*

| **Study** | **Sample**  **Size** | **Number who discontinued treatment** | **Reasons for discontinuation**  (% of all discontinuations) |
| --- | --- | --- | --- |
| **Studies reporting reasons for discontinuations provided directly by patients** | | | |
| Borg Xuereb 2016 (1) | 11 | 3 | One patient who discontinued warfarin did so due to his employer being unsympathetic regarding clinic attendance, and another did so due to regular management interfering with his desire to travel. |
| Gumbinger 2015 (2) | 139  (109 taking at least one dose of OAC treatment) | 21 patients | **Various OAC treatments:**  **Decision made by primary care physician (PCP) (n = 18):**  Bleeding event = 22%  Decline in functional status = 28%  Diagnosis of dementia = 17%  Increased risk of falling = 22%  Follow up ECG without AF = 6%  **Decision made by patient (n = 3):**  Bleeding event = 67% |
| **Studies reporting reasons for discontinuations as selected by a physician from a pre-specified list** | | | |
| Jackson II 2018 (3) | 7,150 | Dabigatran = 169 patients  Warfarin = 1,158 patients | **Dabigatran:**  Physician preference = 13%  Patient refusal = 8.8%  Bleeding events = 6.5%  High risk of bleeding = 3.4%  Gastrointestinal upset = 6.5%  Other = 11.2%  **Warfarin:**  Physician preference = 26.6%  Patient refusal = 14.4%  Bleeding events = 7.1%  High bleeding event = 2%  Gastrointestinal upset = 0.4%  Other = 8.9% |
| O'Brien 2014 (4) | 7,121 | 790 patients  (only 407 reported a reason) | **Warfarin (n = 407):**  Physician preference = 47.7%  Patient refusal = 21.1%  Bleeding event = 20.2%  Frequent falls/frailty = 10.8%  High bleeding risk = 9.8%  Unable to adhere/monitor warfarin = 4.7%  Gastrointestinal upset = 3%  Prior intracerebral haemorrhage = 1.2%  Comorbid illness = 1%  Need for dual antiplatelet therapy = 1%  Allergy = 0.7%  Pregnancy = 0.3%  Occupational risk = 0.3%  Other = 27.3% |
| Paquette 2017 (5) | 2,932 | 437 patients | **Dabigatran:**  Dyspepsia =2.3%  Adverse events = 6.2%  Serious adverse events = 9.8%  Bleeding events = 9.1%  Hypersensitivity to agent = 1.4%  Cost of treatment = 2.3%  Social reason = 1.6%  Bruising = 0%  Bridging therapy start = 1.6%  Dementia = 0.5%  Severe interaction with concomitant medication = 0.2%  Other = 65.1% |
| Paquette 2018 (6) | 4,873 | 687 patients | **Dabigatran:**  Adverse events = 9.4%  Other = 17.5% |
| **Studies reporting reasons for discontinuations as recorded on medical records** | | | |
| Park 2019 (7) | 866 | 59 patients | **Any VKA:**  Unknown = 10%  Uncontrolled INR = 46.3%  Major bleeding = 1.5%  Non-major bleeding = 4%  Interaction with other drugs = 4%  Patient's needs = 21.4%  Stable sinus rhythm (SR) = 8.5%  Switch to NOAC = 1.5%  Comorbidity = 3% |
| Renner 2019 (8) | 319 | 45 patients | **Any OAC:**  Drug cost = 38%  Minor bleeding = 18%  Patient preference = 18%  Side effects other than bleeding = 13%  Thrombosis = 11%  Major bleeding = 2% |
| Naganuma 2017 (9) | 819 | Reasons are from all patients who discontinued or switched treatment    Dabigatran = 72 patients  Rivaroxaban = 37 patients  Apixaban = 30 patients  Warfarin = 22 patients | **Dabigatran:**  Any adverse event = 44.6%  Adverse event: gastrointestinal symptoms = 13.8%  Adverse event: bleeding = 9.7%  Adverse event: abnormal lab data = 12.5%  Adverse event: other = 8.3%  Worsened renal function = 8.3%  Patient desires = 16.6%  Other = 30.4%  **Rivaroxaban:**  Any adverse event = 46%  Adverse event: gastrointestinal symptoms = 13.5%  Adverse event: bleeding = 24.5%  Adverse event: abnormal lab data = 0%  Adverse event: other = 8%  Worsened renal function = 0%  Patient desires = 21.5%  Other = 32.5%  **Apixaban:**  Any adverse event = 56.6%  Adverse event: gastrointestinal symptoms = 6.7%  Adverse event: bleeding = 10.1%  Adverse event: abnormal lab data = 16.8%  Adverse event: other = 23.6%  Worsened renal function = 0%  Patient desires = 10.1%  Other = 33.7%  **Warfarin:**  Any adverse event = 31.8%  Adverse event: gastrointestinal symptoms = 4.8%  Adverse event: bleeding = 13.5%  Adverse event: abnormal lab data = 8.7%  Adverse event: other = 4.8%  Worsened renal function = 0%  Patient desires = 13.5%  Other = 54.9% |
| Shiga 2015 (10) | 601 | Reasons are from all patients who discontinued or switched treatment    Dabigatran = 65 patients  Rivaroxaban = 30 patients  Apixaban = 18 patients  Warfarin = 33 patients | **Dabigatran**  Patient’s own decision = 7.7%  Adverse event: all = 41.6%  Adverse event: gastrointestinal symptoms = 12.4%  Adverse event: bleeding = 4.7%  Adverse event: abnormal laboratory data = 12.4%  Adverse event: other = 12.4%  Worsened renal function = 9.2%  Patient desire = 9.2%  Return to sinus rhythm = 9.2%  Other = 29.2%  **Rivaroxaban**  Patient’s own decision = 13.2%  Adverse event: all = 36.7%  Adverse event: gastrointestinal symptoms = 6.8%  Adverse event: bleeding = 16.8%  Adverse event: abnormal laboratory data = 3.2%  Adverse event: other = 10%  Worsened renal function = 0%  Patient desire = 33.2%  Return to sinus rhythm = 10%  Other = 20%  **Apixaban**  Patient’s own decision = 5.7%  Adverse event: all = 49.9%  Adverse event: gastrointestinal symptoms = 0%  Adverse event: bleeding = 5.7%  Adverse event: abnormal laboratory data = 16.4%  Adverse event: other = 27.8%  Worsened renal function = 0%  Patient desire = 11.3%  Return to sinus rhythm = 16.4%  Other = 22.1%  **Warfarin**  Patient’s own decision = 9.1%  Adverse event: all = 18.2%  Adverse event: gastrointestinal symptoms = 3%  Adverse event: bleeding = 3%  Adverse event: abnormal laboratory data = 9.1%  Adverse event: other = 3%  Worsened renal function = 0%  Patient desire = 9.1%  Poor control of anticoagulation = 6.1%  Return to sinus rhythm = 42.4%  Other = 24.2% |
| Bertozzo 2016 (11) | 798 | 84 patients  (148 patients discontinued or switched) | **Warfarin:**  **Person responsible for the decision (n = 148):**  Specialist advice = 80.4%  GP advice = 12.8%  Patient/caregiver advice = 1.4%  Unknown = 5.4%  **Reason for discontinuation (n = 84):**  Frailty or low life-expectancy = 38%  Bleeding complications = 25%  Sinus rhythm restoration = 29%  Logistic issues = 1%  Low patient compliance = 4%  Unknown = 3% |
| Ho 2014 (12) | 467 | 101 (unclear if this includes patients who switched treatment) | **Dabigatran:**  Patients concerns: all = 11.9%  Patients concerns: dosing frequency = 5.9%  Patients concerns: side effect concerns = 4%  Patients concerns: monitoring concerns = 1%  Patients concerns: financial concerns = 1%  Physician’s choice = 10.9%  Any adverse event = 62.4%  Adverse event: dyspepsia = 30.7%  Adverse event: minor bleeding = 8.9%  Adverse event: major gastrointestinal bleeding = 7.9%  Adverse event: intracranial haemorrhage = 1%  Adverse event: other = 13.9%  Other medical reasons: all = 4%  Other medical reasons: worsening renal function = 3%  Other medical reasons: drug-drug interaction = 3%  Unknown = 8.9% |

NOTE: In many cases patients or physicians could report more than one reason

**References**

1. Borg Xuereb C, Shaw RL, Lane DA. Patients' and physicians' experiences of atrial fibrillation consultations and anticoagulation decision-making: A multi-perspective IPA design. Psychology & health. 2016;31(4):436-55.

2. Gumbinger C, Holstein T, Stock C, Rizos T, Horstmann S, Veltkamp R. Reasons underlying non-adherence to and discontinuation of anticoagulation in secondary stroke prevention among patients with atrial fibrillation. European Neurology. 2015;73(3-4):184-91.

3. Jackson LR, Kim S, Shrader P, Blanco R, Thomas L, Ezekowitz MD, et al. Early therapeutic persistence on dabigatran versus warfarin therapy in patients with atrial fibrillation: results from the Outcomes Registry for Better Informed Treatment of Atrial Fibrillation (ORBIT-AF) registry. Journal of thrombosis and thrombolysis. 2018;46(4):435-9.

4. O'Brien EC, Simon DN, Allen LA, Singer DE, Fonarow GC, Kowey PR, et al. Reasons for warfarin discontinuation in the Outcomes Registry for Better Informed Treatment of Atrial Fibrillation (ORBIT-AF). American heart journal. 2014;168(4):487-94.

5. Paquette M, Riou França L, Teutsch C, Diener HC, Lu S, Dubner SJ, et al. Persistence With Dabigatran Therapy at 2 Years in Patients With Atrial Fibrillation. Journal of the American College of Cardiology. 2017;70(13):1573-83.

6. Paquette M, Huisman MV, Lip GYH, et al. When are atrial fibrillation patients at risk to discontinue anticoagulation treatment? Results from the GLORIA-AF Registry. Eur Heart J 2018; 39 (suppl 1): 1007 [abstr P4779].

7. Park HS, Kim YH, Kim JS, Oh YS, Shin DG, Pak HN, et al. Status of international normalized ratio control and treatment patterns in patients with nonvalvular atrial fibrillation taking vitamin K antagonist with or without antiplatelet therapy: Results from KORAFII registry. Journal of arrhythmia. 2019;35(4):593-601.

8. Renner E, Mouland E, Saad S, Ha N, Kim P. Reasons for discontinuation of direct oral anticoagulant therapy in patients with non-valvular atrial fibrillation. Journal of thrombosis and thrombolysis. 2019;47(4):617-8.

9. Naganuma M, Shiga T, Nagao T, Maruyama K, Suzuki A, Murasaki K, et al. Renal function and treatment persistence with non-Vitamin K antagonist oral anticoagulants in Japanese patients with atrial fibrillation: A single-center experience. Japanese Journal of Clinical Pharmacology and Therapeutics. 2016;47(3):115-22.

10. Shiga T, Naganuma M, Nagao T, Maruyama K, Suzuki A, Murasaki K, et al. Persistence of non-vitamin K antagonist oral anticoagulant use in Japanese patients with atrial fibrillation: A single-center observational study. Journal of arrhythmia. 2015;31(6):339-44.

11. Bertozzo G, Zoppellaro G, Granziera S, Marigo L, Rossi K, Petruzzellis F, et al. Reasons for and consequences of vitamin K antagonist discontinuation in very elderly patients with non-valvular atrial fibrillation. Journal of Thrombosis and Haemostasis. 2016;14(11):2124-31.

12. Ho MH, Ho CW, Cheung E, Chan PH, Hai JJ, Chan KH, et al. Continuation of dabigatran therapy in "real-world" practice in Hong Kong. PLoS ONE. 2014;9(8).
